# GAAD Score as a Prognostic Model for Recurrence of Hepatocellular Carcinoma After Curative Resection

**DOI:** 10.1002/kjm2.70318

**Published:** 2026-11-14

**Authors:** Yueh‐Jen Chung, Chia‐Che Wu, Wei‐Feng Li, Yueh‐Wei Liu, Hsin‐Ming Wang, Yuan‐Hung Kuo, Chao‐Hung Hung, Mu‐Jung Tsai, Ming‐Chao Tsai

**Affiliations:** ^1^ Division of Hepato‐Gastroenterology, Department of Internal Medicine Kaohsiung Chang Gung Memorial Hospital Kaohsiung Taiwan; ^2^ Kaohsiung Municipal Feng Shan Hospital ‐ Under the Management of Chang Gung Medical Foundation Kaohsiung Taiwan; ^3^ Division of Hematology Oncology, Department of Internal Medicine Kaohsiung Chang Gung Memorial Hospital Kaohsiung Taiwan; ^4^ Division of General Surgery, Department of Surgery Kaohsiung Chang Gung Memorial Hospital and Chang Gung University College of Medicine Kaohsiung Taiwan; ^5^ School of Medicine Kaohsiung Medical University Hospital Kaohsiung Taiwan

**Keywords:** AFP, GAAD, hepatocellular carcinoma (HCC), microvascular invasion (MVI), PIVKA‐II

## Abstract

Hepatocellular carcinoma (HCC) has a high recurrence rate after curative resection. The GAAD score, which combines gender, age, α‐fetoprotein (AFP), and PIVKA‐II, was developed for HCC detection, but its prognostic value after resection remains unclear. We retrospectively analyzed 294 patients who underwent curative‐intent liver resection for HCC at a single center. The prognostic associations of preoperative GAAD, AFP, and PIVKA‐II with recurrence‐free survival (RFS) and overall survival (OS) were evaluated using Kaplan–Meier analysis and Cox regression. During a median follow‐up of 86.8 months (IQR, 26.6–112.3), 168 patients (57.1%) developed recurrence and 114 (38.8%) died. GAAD ≥ 1 was associated with shorter RFS (*p* = 0.005) and remained independently associated with recurrence after adjustment for clinicopathological factors (HR 1.85, 95% CI 1.09–3.15; *p* = 0.023). This association remained significant in patients with BCLC stage 0/A. Among patients with AFP < 7 ng/mL, GAAD ≥ 1 remained independently associated with recurrence, whereas PIVKA‐II ≥ 22 ng/mL did not. GAAD ≥ 1 was not associated with OS. In conclusion, GAAD ≥ 1 was independently associated with postoperative HCC recurrence but not with OS. Given its modest discrimination and cohort‐derived cutoff, these exploratory findings require external validation.

**Abbreviations**
AASLD: American Association for the Study of Liver Diseases
AFP: α‐fetoprotein
CT: computed tomography
DCP: des‐carboxyprothrombin
EASL: European Association for the Study of the Liver
ECLIA: electrochemiluminescence immunoassay
GAAD: Gender, age, AFP, and des‐gamma‐carboxy prothrombin
HBV: hepatitis B virus
HCC: Hepatocellular carcinoma
HCV: hepatitis C virus
MRI: magnetic resonance imaging
MVI: microvascular invasion
OS: overall survival
PIVKA‐II: Prothrombin induced by vitamin K absence or antagonist II
RFS: Recurrence‐free survival
ROC: receiver‐operating characteristic

## 1 Introduction

Hepatocellular carcinoma (HCC) is one of the most common malignancies worldwide, and curative‐intent hepatectomy remains a mainstay treatment for selected patients with early‐stage disease [1, 2, 3]. However, even after curative resections, the 5‐year recurrence rate often exceeds 50%, posing a major challenge to long‐term survival [4]. Early identification of patients at high risk of recurrence may facilitate risk‐adapted postoperative surveillance and selection of candidates for adjuvant strategies [5]. Therefore, practical and reliable preoperative prognostic tools for recurrence risk stratification are clinically needed.

AFP and PIVKA‐II are widely used serum biomarkers for HCC and provide complementary clinical information. PIVKA‐II may be particularly useful in patients with low or normal AFP levels, in whom AFP alone has limited sensitivity [6, 7, 8]. Beyond their diagnostic roles, both biomarkers have been associated with tumor aggressiveness and postoperative outcomes, including recurrence after curative resection [9, 10, 11, 12]. These findings provide a rationale for combining AFP and PIVKA‐II with clinical variables to improve risk assessment in patients with HCC.

The gender, age, AFP, and des‐gamma‐carboxy prothrombin (GAAD) algorithm is a non‐invasive multivariable score that integrates gender and age with serum AFP and PIVKA‐II [13, 14]. GAAD was originally developed for HCC detection and has demonstrated good diagnostic performance, including for early‐stage HCC [15, 16]. However, its prognostic value after curative resection has not been established. Because GAAD incorporates biomarkers associated with tumor aggressiveness and recurrence risk, we hypothesized that it may also provide prognostic information in the postoperative setting. Therefore, this study aimed to evaluate the association of preoperative GAAD with recurrence‐free survival and overall survival in patients with HCC undergoing curative‐intent liver resection.

## 2 Methods

### 2.1 Study Design

This retrospective cohort study was conducted at Kaohsiung Chang Gung Memorial Hospital (KCGMH), a tertiary medical center in southern Taiwan. The study protocol was approved by the Institutional Review Board of KCGMH (approval number: 202400435B0C501), which waived the requirement for informed consent because of the retrospective nature of the study.

### 2.2 Patients

A retrospective review of the KCGMH HCC registry was performed for the period from January 1, 2010, to December 31, 2019. Eligible patients were aged ≥ 20 years and had undergone curative‐intent hepatic resection, defined as complete macroscopic tumor removal with no gross residual intrahepatic or extrahepatic disease and a microscopically tumor‐free resection margin (R0 resection). Patients with extrahepatic metastasis and macrovascular invasion identified on preoperative imaging were not considered candidates for curative‐intent resection. Surgical margin width was measured on the resected specimen. Patients with incomplete clinical data or a follow‐up duration of less than 3 months were excluded. Baseline demographic data, serum biochemistry, and tumor pathological characteristics were extracted from the registry. Histopathological evaluation included assessment for satellite nodules, microvascular invasion (MVI), and tumor differentiation, graded according to the modified nuclear grading system of Edmondson and Steiner [17]. The degree of hepatic fibrosis in non‐tumorous liver tissue was assessed using the Ishak classification, with liver cirrhosis defined as a fibrosis score of 5–6 [18]. Hepatic steatosis was defined histologically as ≥ 5% steatotic hepatocytes.

Chronic hepatitis B (CHB) was defined as seropositivity for hepatitis B surface antigen (HBsAg) for at least 6 months, and chronic hepatitis C (CHC) as a positive anti‐HCV antibody with detectable HCV RNA or a previously confirmed chronic HCV infection. Patients with both infections were classified as CHB + CHC, and those negative for both as non‐B, non‐C (NBNC). Antiviral therapy in patients with HBV‐related HCC was defined as nucleos(t)ide analogue therapy initiated before hepatectomy, whereas antiviral therapy in patients with HCV‐related HCC was defined as interferon‐based or direct‐acting antiviral therapy resulting in documented sustained virologic response.

RFS was defined as the interval from the date of hepatectomy to the first documented HCC recurrence or last follow‐up. OS was defined as the interval from the date of hepatectomy to death from any cause or last follow‐up. Tumor recurrence was based on characteristic features on contrast‐enhanced computed tomography (CT) or magnetic resonance imaging (MRI), following the guidelines of the European Association for the Study of the Liver (EASL) and the American Association for the Study of Liver Diseases (AASLD) [4, 19]. In the absence of histological confirmation, imaging criteria alone were considered sufficient.

### 2.3 Measurement of GAAD Score

Preoperative serum AFP and PIVKA‐II levels were measured in stored serum samples obtained from the tissue and biobank of KCGMH. AFP and PIVKA‐II were measured using the Elecsys AFP and Elecsys PIVKA‐II assays, respectively (Roche Diagnostics International Ltd., Rotkreuz, Switzerland), on a Cobas e 801 analyzer according to the manufacturer's instructions. Results were expressed in nanograms per milliliter (ng/mL). Laboratory personnel performing the assays were blinded to all clinical and pathological data to avoid measurement bias.

The GAAD algorithm incorporates four variables: gender, age, serum AFP, and serum PIVKA‐II levels. The development and validation of the GAAD algorithm have been previously described [15, 16]. In brief, GAAD was derived from a logistic regression model trained on a large, multicenter dataset and remains proprietary; the scoring formula has not been published and can only be calculated via the Elecsys GAAD platform provided by Roche Diagnostics. In this study, GAAD scores were generated using the manufacturer's tool, which automatically integrates the AFP and PIVKA‐II assay results with demographic variables.

### 2.4 Statistical Analysis

Continuous variables are reported as medians with interquartile ranges (IQR), while categorical variables are summarized as frequencies and percentages. Baseline characteristics were compared between patients with and without recurrence. Differences between groups were evaluated using the Mann–Whitney U test for continuous variables and the Chi‐square test or Fisher's exact test for categorical variables, as applicable. Receiver operating characteristic (ROC) curve analysis was performed to evaluate the ability of GAAD, AFP, and PIVKA‐II to discriminate between patients with and without HCC recurrence. The area under the ROC curve (AUROC) with 95% confidence intervals (CIs) was calculated for each marker. The optimal cutoff value was determined by maximizing the Youden index. For clinical interpretability, the resulting cutoff values were rounded to practical thresholds for subsequent analyses. RFS and OS were estimated using the Kaplan–Meier method and compared using the log‐rank test. Hazard ratios (HRs) with 95% CIs were estimated using Cox proportional hazards regression. Variables with a univariable *p*‐value < 0.20 were considered for multivariable analysis. Because GAAD is mathematically derived from sex, age, AFP, and PIVKA‐II, two separate multivariable models were constructed to avoid collinearity: model 1 (GAAD model) included GAAD, liver cirrhosis, BCLC stage, tumor size, tumor number, MVI, satellite nodules, and antiviral therapy; model 2 (conventional biomarker model) included age, sex, diabetes mellitus, AFP, PIVKA‐II, and the same clinicopathological covariates as model 1, without GAAD. Collinearity among sex, age, AFP, PIVKA‐II, and GAAD was evaluated using Spearman correlation coefficients. To address confounding by tumor stage, recurrence analyses were repeated in patients with BCLC stage 0/A. The prognostic value of GAAD was further examined in patients with low AFP (< 7 ng/mL), using the same univariable screening and two multivariable models, and in those with both low AFP and PIVKA‐II (< 22 ng/mL) using Kaplan–Meier analysis. Treatments after recurrence were extracted from medical records and classified as curative‐intent local treatment (repeat resection, radiofrequency ablation, or percutaneous ethanol injection), transarterial chemoembolization or radiotherapy, systemic therapy (immunotherapy, targeted therapy, or chemotherapy), and palliative care or unknown. Statistical significance was defined as a two‐tailed *p*‐value of < 0.05.

## 3 Results

### 3.1 Patient Characteristics

Baseline characteristics are summarized in Table 1. The median age was 63.0 years (IQR, 54.0–70.0), and 229 patients (77.9%) were male. Regarding etiology, 152 patients (51.7%) had CHB, 83 (28.2%) had CHC, 19 (6.5%) had both CHB and CHC, and 40 (13.6%) were classified as NBNC. Antiviral therapy was received by 83 of 171 (48.5%) patients with HBV‐related HCC and 70 of 102 (68.6%) patients with HCV‐related HCC. Liver cirrhosis was present in 114 patients (38.9%). According to the BCLC staging system, 20 patients (6.8%) had stage 0 disease, 217 (73.8%) stage A, 34 (11.6%) stage B, and 23 (7.8%) stage C. The median AFP level was 8.0 ng/mL (IQR, 3.1–124.0), and 153 patients (52.0%) had AFP ≥ 7 ng/mL. The median PIVKA‐II level was 49.5 ng/mL (IQR, 20.5–284.0), and 210 patients (71.4%) had PIVKA‐II ≥ 22 ng/mL. Forty‐six patients (15.6%) had both AFP < 7 ng/mL and PIVKA‐II < 22 ng/mL. The median GAAD score was 5.5 (IQR, 1.6–9.5), and 245 patients (83.3%) had GAAD ≥ 1.

**TABLE 1:** Characteristics of the HCC patients who underwent curative resection.

	Total (*n* = 294)	Recurrence (*n* = 168)	Non‐recurrence (*n* = 126)	*p*
Age (years), median (IQR)	63.0 (54.0–70.0)	63.0 (55.1–70.0)	61.0 (53.0–69.0)	0.181
Male, *n* (%)	229 (77.9)	136 (81.0)	93 (73.8)	0.144
Diabetes mellitus, *n* (%)	91 (31.0)	47 (28.0)	44 (34.9)	0.202
Alcoholism, *n* (%)	35 (11.9)	22 (13.1)	13 (10.3)	0.552
Etiology				0.004
CHB, *n* (%)	152 (51.7)	78 (46.4)	74 (58.7)	
CHC, *n* (%)	83 (28.2)	61 (36.3)	22 (17.5)	
CHB + CHC	19 (6.5)	11 (6.5)	8 (6.3)	
NBNC	40 (13.6)	18 (10.7)	22 (17.5)	
Anti‐HBV treatment	83 (48.5)	46 (51.7)	37 (45.1)	0.391
Anti‐HCV treatment	70 (68.6)	48 (66.7)	22 (73.3)	0.509
Platelets < 150 × 10^3^/μL, *n* (%)	111 (37.8)	71 (42.3)	40 (31.7)	0.066
Albumin (g/dL), median (IQR)	4.1 (3.8–4.4)	4.0 (3.7–4.3)	4.3 (4.0–4.5)	< 0.001
Total bilirubin (mg/dL), median (IQR)	0.7 (0.5–0.9)	0.7 (0.5–0.9)	0.7 (0.6–0.9)	0.268
AFP (ng/mL)	8.0 (3.1–124.0)	9.3 (3.3–182.0)	5.5 (2.8–90.7)	0.082
PIVKA‐II (ng/mL)	49.5 (20.5–284.0)	54.9 (22.0–327.3)	45.6 (18.1–227.3)	0.214
GAAD, median (IQR)	5.5 (1.6–9.5)	6.1 (2.2–9.7)	4.8 (1.0–9.2)	0.028
Ishak score, median (IQR)	3.0 (2.0–6.0)	4.0 (2.0–6.0)	3.0 (1.0–5.0)	0.012
Liver cirrhosis, *n* (%)	114 (38.9)	75 (44.9)	39 (31.0)	0.015
BCLC stage, *n* (%)				0.043
0	20 (6.8)	11 (6.5)	9 (7.1)	
A	217 (73.8)	116 (69.0)	101 (80.2)	
B	34 (11.6)	22 (13.1)	12 (9.5)	
C	23 (7.8)	19 (11.3)	4 (3.2)	
Tumor size (cm)^a^, median (IQR)	3.5 (2.4–5.3)	3.5 (2.5–5.7)	3.3 (2.3–5.0)	0.304
Tumor number, single, *n* (%)	249 (84.7)	140 (83.3)	109 (86.5)	0.454
MVI, *n* (%)	158 (53.7)	103 (61.3)	55 (43.7)	0.003
Satellite nodule, *n* (%)	17 (5.8)	12 (7.1)	5 (4.0)	0.248
Hepatic steatosis, *n* (%)	82 (28.2)	41 (24.8)	41 (32.5)	0.148
Resection margin (mm), median (IQR)	10.0 (4.0–15.0)	9.0 (4.0–15.0)	10.0 (5.0–15.0)	0.461
Death, *n* (%)	114 (38.8)	86 (51.2)	28 (22.2)	< 0.001
Follow‐up (months), median (IQR)	86.8 (26.6–112.3)	83.0 (27.6–112.5)	90.9 (26.4–112.7)	0.539

*Note:* Data are expressed as median (interquartile range) or *n* (%). *p* values were calculated with the Mann–Whitney *U* test for continuous variables and the Chi‐square test or Fisher's exact test for categorical variables.

Abbreviations: AFP, alpha‐fetoprotein; BCLC, Barcelona clinical liver cancer; CHB, chronic hepatitis B; CHC, chronic hepatitis C; NBNC, non‐B, non‐C; PIVKA‐II, protein induced by vitamin K absence or antagonist II; MVI, microvascular invasion.

^a^ Diameter of the largest tumor nodule.

During a median follow‐up of 86.8 months (IQR, 26.6–112.3), 168 patients (57.1%) developed HCC recurrence and 114 (38.8%) died. Compared with patients without recurrence, those with recurrence had lower serum albumin levels (*p* < 0.001), higher Ishak fibrosis scores (*p* = 0.012), a higher prevalence of liver cirrhosis (44.9% vs. 31.0%, *p* = 0.015), and a higher prevalence of MVI (61.3% vs. 43.7%, *p* = 0.003). BCLC stage and HCC etiology distributions also differed significantly between the two groups (*p* = 0.043 and *p* = 0.004, respectively), with CHC being more frequent among patients with recurrence (36.3% vs. 17.5%). Antiviral treatment rates did not differ significantly between the groups (HBV, *p* = 0.391; HCV, *p* = 0.509). AFP and PIVKA‐II levels were also comparable between the groups, whereas the median GAAD score was significantly higher in patients with recurrence than in those without recurrence (6.1 vs. 4.8, *p* = 0.028; Table 1).

### 3.2 Prognostic Cutoffs and Associations Between GAAD and HCC Outcomes

ROC analysis identified optimal prognostic cutoffs of ≥ 1 for GAAD (AUC = 0.675; sensitivity = 86.3%, specificity = 29.4%). AUROCs of 0.559 (95% CI, 0.493–0.626) and 0.542 (95% CI, 0.475–0.610) for AFP and PIVKA‐II, respectively. The optimal cutoff values were 7 for AFP and 22 for PIVKA‐II, corresponding to sensitivities of 58.3% and 79.8% and specificities of 55.6% and 34.1%, respectively. For subsequent statistical analyses, the cutoff values were rounded to clinically practical thresholds of AFP ≥ 7 ng/mL and PIVKA‐II ≥ 22 ng/mL to facilitate interpretation and clinical applicability. Accordingly, 245 patients (83.3%) had GAAD ≥ 1 and 49 (16.7%) had GAAD < 1.

In the whole cohort, patients with GAAD ≥ 1, AFP ≥ 7 ng/mL, and PIVKA‐II ≥ 22 ng/mL all had significantly shorter RFS (*p* = 0.005, 0.021, and 0.022, respectively; Figure 1A–C). In contrast, GAAD, AFP, and PIVKA‐II were not associated with OS (Figure 1D–F).

**FIGURE 1:**
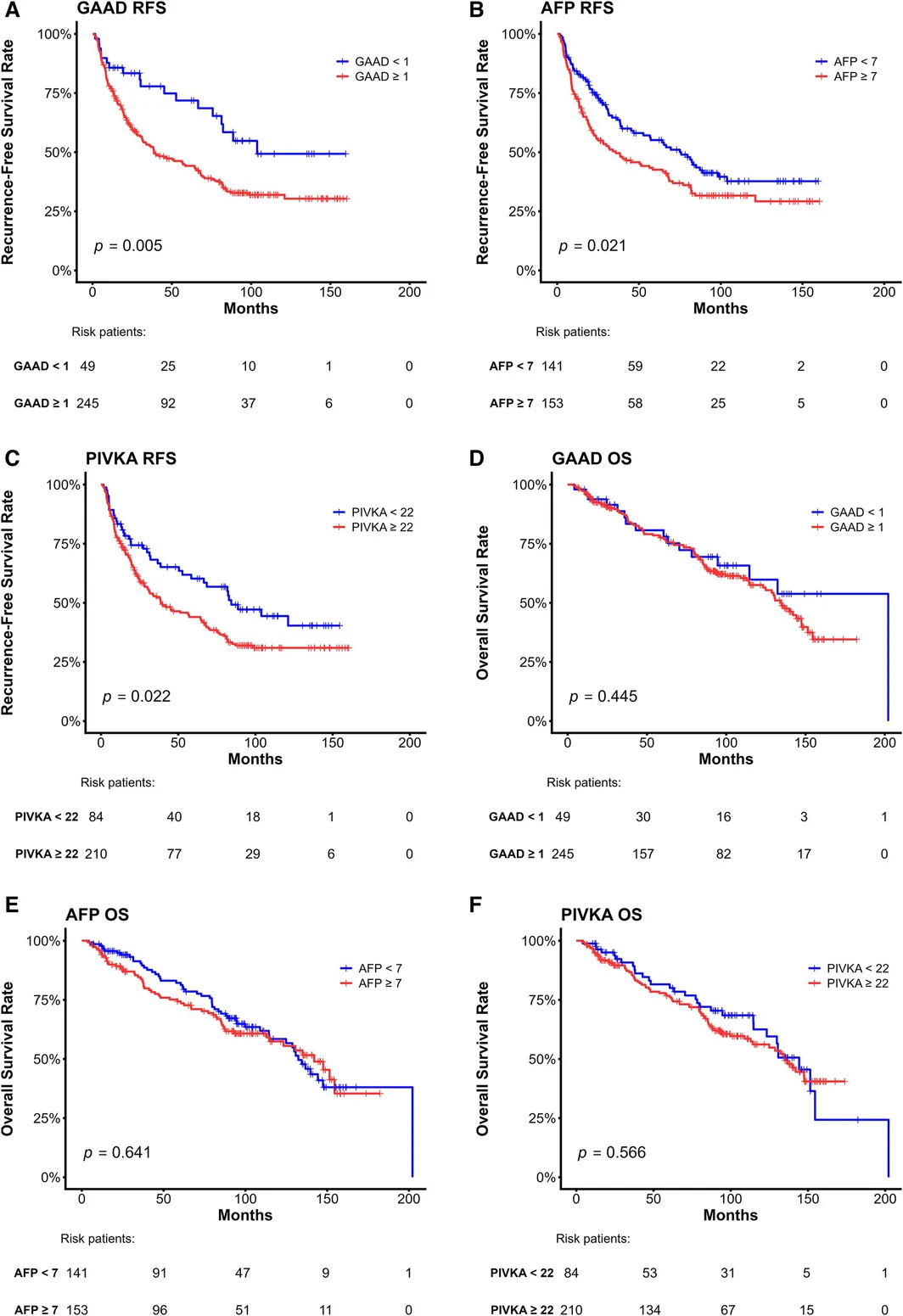
Recurrence‐free survival (RFS; A–C) and overall survival (OS; D–F) among patients with hepatocellular carcinoma (HCC) after curative resection, stratified by (A, D) GAAD score, (B, E) AFP level, and (C, F) PIVKA‐II level.

### 3.3 Prognostic Factors Associated With HCC Recurrence

In univariable Cox regression analysis (Table 2), GAAD ≥ 1 was significantly associated with an increased risk of recurrence (HR 1.99, 95% CI 1.22–3.25; *p* = 0.006). AFP ≥ 7 ng/mL, PIVKA‐II ≥ 22 ng/mL, liver cirrhosis, BCLC stage B/C, tumor size ≥ 5 cm, MVI, and satellite nodules were also significantly associated with recurrence.

**TABLE 2:** Prognostic factors associated with HCC recurrence.

Variable	Comparison	Univariable		Multivariable 1		Multivariable 2	
		HR (95% CI)	*p*	HR (95% CI)	*p*	HR (95% CI)	*p*
Age (years)	> 60 versus ≦ 60	1.29 (0.94–1.77)	0.114			1.54 (1.09–2.19)	0.016
Sex	Male versus Female	1.33 (0.91–1.96)	0.145			1.42 (0.94–2.16)	0.100
Diabetes mellitus	Yes versus No	0.92 (0.65–1.28)	0.605				
AFP (ng/mL)	≧ 7 versus < 7	1.43 (1.05–1.94)	0.022			1.12 (0.79–1.59)	0.522
PIVKA‐II (ng/mL)	≧ 22 versus < 22	1.52 (1.06–2.17)	0.023			1.44 (0.96–2.17)	0.082
GAAD	≧ 1 versus < 1	1.99 (1.22–3.25)	0.006	1.85 (1.09–3.15)	0.023		
Liver cirrhosis	Yes versus No	1.52 (1.12–2.07)	0.007	1.45 (1.03–2.02)	0.031	1.41 (1.00–2.00)	0.050
BCLC stage	B/C versus 0/A	2.10 (1.47–2.99)	< 0.001	2.99 (1.56–5.72)	< 0.001	3.30 (1.74–6.24)	< 0.001
Tumor size (cm)	≧ 5 versus < 5	1.44 (1.04–1.99)	0.029	1.22 (0.83–1.80)	0.317	1.12 (0.74–1.68)	0.601
Tumor number	Multiple versus Single	1.46 (0.96–2.21)	0.080	2.25 (1.08–4.69)	0.031	2.68 (1.29–5.58)	0.008
MVI	Yes versus No	1.87 (1.37–2.55)	< 0.001	1.46 (1.03–2.07)	0.032	1.52 (1.07–2.16)	0.020
Satellite nodule	Yes versus No	2.49 (1.38–4.48)	0.002	1.03 (0.49–2.16)	0.945	1.45 (0.69–3.03)	0.328
Antiviral therapy	Yes versus No	0.75 (0.54–1.04)	0.081	0.78 (0.56–1.09)	0.139	0.86 (0.61–1.21)	0.385

*Note:* Multivariable 1: include GAAD, liver cirrhosis, BCLC stage, tumor size, tumor number, MVI, satellite nodule, and antiviral therapy. Multivariable 2: include age, sex, diabetes mellitus, AFP, PIVKA‐II, liver cirrhosis, BCLC stage, tumor size, tumor number, MVI, satellite nodule, and antiviral therapy.

Abbreviations: AFP, alpha‐fetoprotein; BCLC, Barcelona clinical liver cancer; CI, confidence interval; HR, hazard ratio; MVI, microvascular invasion; PIVKA‐II, protein induced by vitamin K absence or antagonist II.

GAAD was strongly correlated with AFP (Spearman *ρ* = 0.723) and PIVKA‐II (*ρ* = 0.672), whereas correlations among gender, age, AFP, and PIVKA‐II were weak (|*ρ*| ≤ 0.187; Supplementary Table S2). Because GAAD is mathematically derived from these components, GAAD and its individual components were analyzed in separate multivariable models. In multivariable model 1, GAAD ≥ 1 remained independently associated with recurrence (HR 1.85, 95% CI 1.09–3.15; *p* = 0.023), together with liver cirrhosis, BCLC stage B/C, multiple tumors, and MVI. In model 2, which included the individual components of GAAD instead of the composite score, age > 60 years, BCLC stage B/C, multiple tumors, and MVI were independently associated with recurrence, whereas AFP ≥ 7 ng/mL and PIVKA‐II ≥ 22 ng/mL were not (Table 2).

### 3.4 Prognostic Factors Associated With HCC Mortality

In univariable Cox regression analysis (Table 3), age > 60 years, BCLC stage B/C, tumor size ≥ 5 cm, MVI, and satellite nodules were significantly associated with mortality. In contrast, GAAD ≥ 1 (HR 1.24, 95% CI 0.72–2.13; *p* = 0.446), AFP ≥ 7 ng/mL, and PIVKA‐II ≥ 22 ng/mL were not significantly associated with mortality. In multivariable model 1, GAAD ≥ 1 was not significantly associated with OS, whereas BCLC stage B/C and satellite nodules remained independently associated with poorer OS. In model 2, age > 60 years, BCLC stage B/C, and satellite nodules were independently associated with poorer OS (Table 3).

**TABLE 3:** Prognostic factors associated with HCC mortality.

Variable	Comparison	Univariable		Multivariable 1		Multivariable 2	
		HR (95% CI)	*p*	HR (95% CI)	*p*	HR (95% CI)	*p*
Age (years)	> 60 versus ≦ 60	2.06 (1.34–3.16)	< 0.001			2.29 (1.43–3.65)	< 0.001
Sex	Male versus Female	1.08 (0.68–1.70)	0.753			1.38 (0.82–2.35)	0.230
Diabetes mellitus	Yes versus No	1.36 (0.93–1.99)	0.119	1.25 (0.80–1.93)	0.325	1.22 (0.77–1.92)	0.402
AFP (ng/mL)	≧ 7 versus < 7	1.09 (0.76–1.58)	0.641			0.97 (0.63–1.51)	0.906
PIVKA‐II (ng/mL)	≧ 22 versus < 22	1.13 (0.74–1.72)	0.566			0.89 (0.55–1.47)	0.660
GAAD	≧ 1 versus < 1	1.24 (0.72–2.13)	0.446	1.23 (0.67–2.26)	0.490		
Liver cirrhosis	Yes versus No	1.34 (0.93–1.94)	0.121	1.03 (0.66–1.59)	0.914	1.01 (0.65–1.57)	0.978
BCLC stage	B/C versus 0/A	2.12 (1.41–3.20)	< 0.001	2.14 (1.10–4.15)	0.025	2.66 (1.34–5.28)	0.005
Tumor size (cm)	≧ 5 versus < 5	1.76 (1.20–2.59)	0.004	1.56 (0.99–2.46)	0.053	1.51 (0.94–2.43)	0.090
Tumor number	Multiple versus Single	1.29 (0.81–2.08)	0.283	1.50 (0.71–3.19)	0.289	1.89 (0.87–4.09)	0.107
MVI	Yes versus No	1.77 (1.21–2.58)	0.003	1.46 (0.93–2.30)	0.102	1.42 (0.90–2.25)	0.137
Satellite nodule	Yes versus No	5.57 (3.10–9.99)	< 0.001	3.22 (1.59–6.52)	0.001	4.25 (2.04–8.86)	< 0.001
Antiviral therapy	Yes versus No	0.95 (0.64–1.40)	0.777	0.95 (0.63–1.44)	0.809	0.91 (0.59–1.39)	0.652

*Note:* Multivariable 1: include GAAD, liver cirrhosis, BCLC stage, tumor size, tumor number, MVI, satellite nodule, and antiviral therapy. Multivariable 2: include age, sex, diabetes mellitus, AFP, PIVKA‐II, liver cirrhosis, BCLC stage, tumor size, tumor number, MVI, satellite nodule, and antiviral therapy.

Abbreviations: AFP, alpha‐fetoprotein; BCLC, Barcelona Clinical Liver Cancer; CI, confidence interval; HR, hazard ratio; MVI, microvascular invasion; PIVKA‐II, protein induced by vitamin K absence or antagonist II.

### 3.5 Subgroup Analysis in BCLC Stage 0/A

Among the 237 patients with BCLC stage 0/A HCC, GAAD ≥ 1 (*p* = 0.012) and AFP ≥ 7 ng/mL (*p* = 0.033) were associated with shorter RFS, whereas PIVKA‐II ≥ 22 ng/mL was not (*p* = 0.085; Figure 2). In multivariable model 1, GAAD ≥ 1 remained independently associated with recurrence (HR 1.82, 95% CI 1.03–3.21; *p* = 0.039), together with liver cirrhosis and MVI (Table 4). In model 2, neither AFP ≥ 7 ng/mL nor PIVKA‐II ≥ 22 ng/mL was significantly associated with recurrence.

**FIGURE 2:**
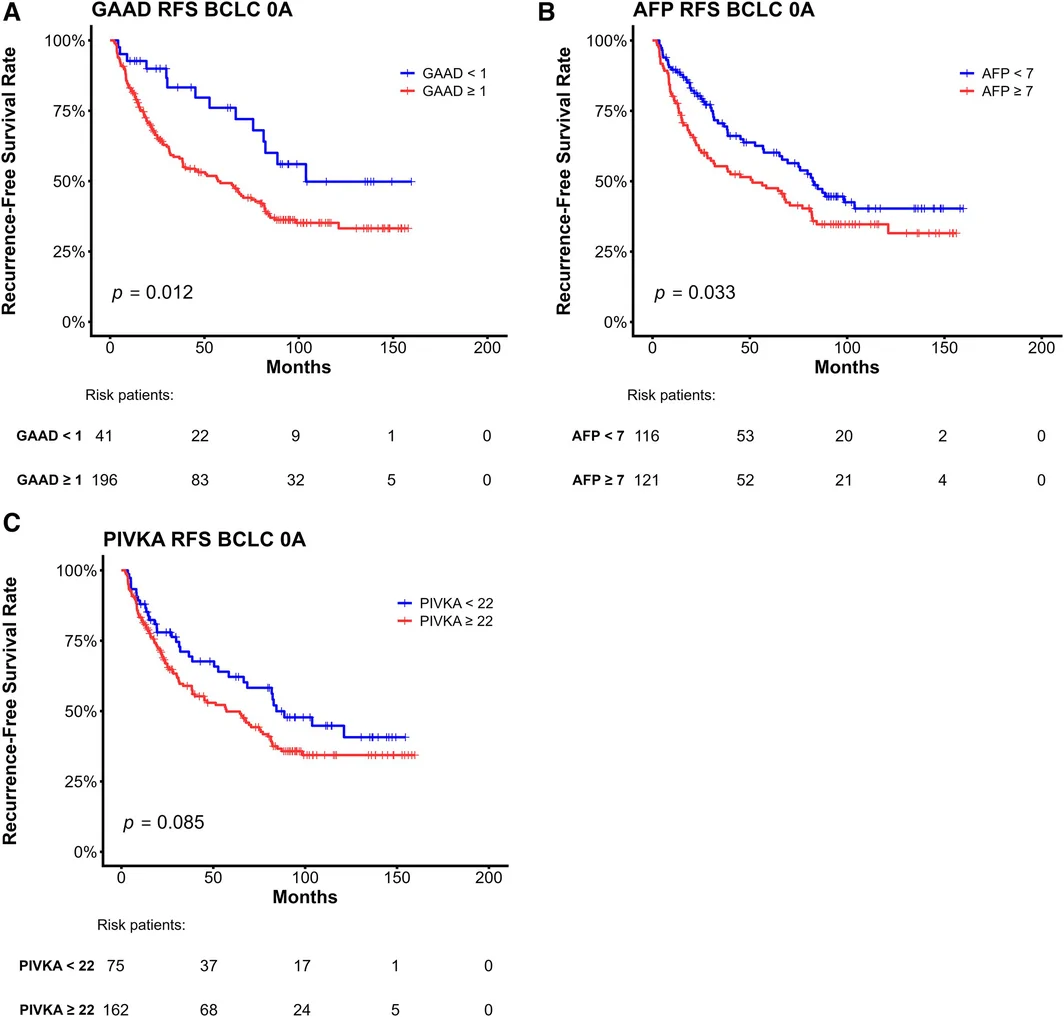
Recurrence‐free survival (RFS) among patients with BCLC stage 0/A hepatocellular carcinoma (HCC) after curative resection, stratified by (A) GAAD score, (B) AFP level, and (C) PIVKA‐II level.

**TABLE 4:** Prognostic factors associated with HCC recurrence in BCLC stage 0/A subgroup.

Variable	Comparison	Univariable		Multivariable 1		Multivariable 2	
		HR (95% CI)	*p*	HR (95% CI)	*p*	HR (95% CI)	*p*
Age (years)	> 60 versus ≦ 60	1.31 (0.91–1.89)	0.147			1.45 (0.99–2.11)	0.055
Sex	Male versus Female	1.10 (0.73–1.67)	0.640			1.16 (0.75–1.79)	0.512
Diabetes mellitus	Yes versus No	1.10 (0.76–1.60)	0.620				
AFP (ng/mL)	≧ 7 versus < 7	1.47 (1.03–2.08)	0.034			1.30 (0.89–1.88)	0.173
PIVKA‐II (ng/mL)	≧ 22 versus < 22	1.41 (0.95–2.09)	0.087			1.32 (0.87–1.99)	0.170
GAAD	≧ 1 versus < 1	2.01 (1.16–3.51)	0.014	1.82 (1.03–3.21)	0.039		
Liver cirrhosis	Yes versus No	1.48 (1.04–2.11)	0.028	1.49 (1.04–2.14)	0.030	1.42 (0.98–2.06)	0.063
Tumor size (cm)	≧ 5 versus < 5	1.11 (0.73–1.68)	0.629				
Tumor number	Multiple versus Single	0.52 (0.13–2.10)	0.358				
MVI	Yes versus No	1.71 (1.20–2.43)	0.003	1.51 (1.05–2.17)	0.026	1.53 (1.05–2.21)	0.025
Satellite nodule	Yes versus No	3.63 (1.48–8.94)	0.005	2.17 (0.85–5.54)	0.105	2.27 (0.87–5.88)	0.093
Antiviral therapy	Yes versus No	0.82 (0.65–1.39)	0.812				

*Note:* Multivariable 1: include GAAD, liver cirrhosis, tumor size, tumor number, MVI, satellite nodule, and antiviral therapy. Multivariable 2: include age, sex, diabetes mellitus, AFP, PIVKA‐II, liver cirrhosis, tumor size, tumor number, MVI, satellite nodule, and antiviral therapy.

Abbreviations: AFP, alpha‐fetoprotein; BCLC, Barcelona Clinical Liver Cancer; CI, confidence interval; HR, hazard ratio; MVI, microvascular invasion; PIVKA‐II, protein induced by vitamin K absence or antagonist II.

### 3.6 Subgroup Analysis in Patients With AFP < 7 Ng/mL

Among the 141 patients with AFP < 7 ng/mL, GAAD ≥ 1, but not PIVKA‐II ≥ 22 ng/mL, was significantly associated with recurrence in univariable Cox regression analysis. In multivariable model 1, GAAD ≥ 1 remained independently associated with recurrence (HR 2.50, 95% CI 1.39–4.51; *p* = 0.002), together with liver cirrhosis and BCLC stage B/C (Supplementary Table S1). In model 2, PIVKA‐II ≥ 22 ng/mL was not significantly associated with recurrence. Notably, among the 46 patients with both AFP < 7 ng/mL and PIVKA‐II < 22 ng/mL, GAAD ≥ 1 was not significantly associated with RFS (*p* = 0.236) or OS (*p* = 0.101; Supplementary Figure S1).

### 3.7 Clinical Characteristics by GAAD Score

Supplementary Table S3 summarizes the clinical and pathological characteristics of patients stratified by GAAD score (< 1 vs. ≥ 1). As expected from the components of the GAAD algorithm, patients with GAAD ≥ 1 were older and had higher AFP and PIVKA‐II levels than those with GAAD < 1 (all *p* < 0.001). In addition, patients with GAAD ≥ 1 had larger tumors and a higher prevalence of MVI. The distributions of gender, HCC etiology, liver cirrhosis, BCLC stage, tumor number, and satellite nodules did not differ significantly between the two groups.

### 3.8 Treatment After Recurrence

Among the 168 patients with recurrence, 82 (48.8%) received curative‐intent local treatment, 52 (31.0%) TACE or radiotherapy, 26 (15.5%) systemic therapy, and 8 (4.8%) palliative care or unknown treatment. The proportion receiving curative‐intent local treatment did not differ between patients with GAAD ≥ 1 and < 1 (50.0% vs. 38.9%, *p* = 0.458). Median post‐recurrence survival was 109.8, 50.0, 27.3, and 2.5 months, respectively, and did not differ by GAAD.

## 4 Discussion

The GAAD score—originally developed as a non‐invasive diagnostic algorithm for HCC screening—integrates gender, age, AFP, and PIVKA‐II. To date, it has not been evaluated as a prognostic tool in the postoperative setting. This study is the first to assess GAAD value in predicting recurrence‐free and overall survival after curative‐intent liver resection for HCC, and we found that a GAAD score ≥ 1 was associated with shorter RFS and remained independently associated with recurrence after adjustment for clinicopathological factors, including in patients with BCLC stage 0/A. However, GAAD showed no association with OS.

In our cohort, AFP ≥ 7 ng/mL and PIVKA‐II ≥ 22 ng/mL were each associated with shorter RFS in univariable analysis but were not independently associated with recurrence in the multivariable model that included the individual components of GAAD. In contrast, GAAD ≥ 1 remained independently associated with recurrence in the model incorporating the composite score. Because GAAD is mathematically derived from gender, age, AFP, and PIVKA‐II and was strongly correlated with AFP and PIVKA‐II (Supplementary Table S2), GAAD and its components were analyzed in separate models. Therefore, we cannot conclude that GAAD is superior to its individual components because their discriminative performance was not formally compared. Furthermore, part of the prognostic signal of GAAD may be attributable to age, which was independently associated with recurrence in the model incorporating the individual GAAD components.

GAAD ≥ 1 was associated with more aggressive tumor features, including larger tumor size and a higher rate of MVI (58.4% vs. 30.6%). This finding is consistent with previous studies showing that PIVKA‐II is related to MVI [20], and MVI is also a well‐known predictor of recurrence and survival after liver resection [21]. In our analysis, GAAD remained associated with recurrence even after adjustment for MVI, BCLC stage, tumor size, and tumor number. This suggests that the prognostic value of GAAD may not be completely explained by these tumor characteristics. However, the effect of other unmeasured tumor‐related factors cannot be excluded. Previous studies have suggested that early recurrence is mainly related to tumor factors, while late recurrence may be more affected by the underlying liver disease and type of treatment rendered for the recurrence [22]. Therefore, because GAAD is mainly based on tumor markers, it may be more useful for predicting tumor‐related recurrence than long‐term mortality.

The lack of association between GAAD and OS may partly reflect the substantial influence of post‐recurrence treatment. After liver resection, overall survival depends heavily on treatment after recurrence [22]. In our cohort, patients who received curative‐intent local treatment after recurrence had a much longer median post‐recurrence survival (109.8 months) than those who received TACE or radiotherapy (50.0 months), systemic therapy (27.3 months), or palliative care (2.5 months). Thus, OS after resection may be influenced not only by baseline tumor characteristics but also by subsequent treatment and disease course.

The type of treatment and post‐recurrence survival did not differ by GAAD, so differences in post‐recurrence treatment do not appear to explain the lack of association between GAAD and OS, although the heterogeneity of treatment may still have diluted an effect. OS is also affected by patient age and non‐tumor‐related death; in our cohort, 68 of 114 deaths (59.6%) were HCC‐related, and age > 60 years and satellite nodules were the main factors associated with mortality. In addition, systemic therapy was used more often in the later part of the study period (11.9% before 2015 vs. 33.3% after 2016), although the number of recurrences in the later period was small. The number of deaths (*n* = 114) may also have limited the power of the OS analysis. Based on these findings, we cannot conclude that GAAD is a useful predictor of OS.

In the BCLC 0/A subgroup, GAAD ≥ 1 remained significantly associated with recurrence (HR 1.82, 95% CI 1.03–3.21), while AFP and PIVKA‐II were not independently associated with recurrence. This finding may be clinically relevant because patients with early‐stage HCC are an important group for decisions regarding surveillance after resection and possible adjuvant treatment. However, this result should be interpreted with caution because it was based on subgroup analysis and the confidence interval was relatively wide. Therefore, this finding should be considered hypothesis‐generating and requires further validation in larger studies.

Previous studies have shown that the sensitivity of AFP for detecting HCC is approximately 40%–50%, meaning that a substantial proportion of patients present with normal AFP levels at diagnosis [23, 24]. The reported sensitivity of PIVKA‐II varies across studies but is generally higher than AFP, ranging from 48% to over 60%, depending on the cutoff used and the population studied [25, 26]. It is also well established that AFP and PIVKA‐II levels are not strongly correlated—patients with normal AFP may still have elevated PIVKA‐II, and vice versa [27, 28]. A subgroup of patients therefore has low levels of both markers, in whom conventional markers provide little basis for recurrence risk stratification. We examined whether GAAD could help in this group. In the 46 patients with low AFP and PIVKA‐II, however, GAAD ≥ 1 was not significantly associated with RFS or OS. These results do not support the use of GAAD for risk stratification in patients with low AFP and PIVKA‐II, although the small sample size means that a modest association cannot be excluded. In these patients, GAAD may largely reflect age and sex, because AFP and PIVKA‐II contribute little, which may limit the additional information it provides.

In the subgroup of patients with AFP < 7 ng/mL, GAAD ≥ 1 remained independently associated with recurrence, whereas PIVKA‐II ≥ 22 ng/mL did not. This subgroup finding is exploratory. GAAD and PIVKA‐II classified 82% of these patients identically; only 26 patients were classified differently, and we did not formally compare the two markers. We therefore cannot conclude that GAAD adds information beyond PIVKA‐II. Its practical advantage is that it combines sex, age, AFP, and PIVKA‐II into one score generated from tests already ordered in HCC care, but this needs to be confirmed in independent cohorts.

Antiviral therapy and etiology are important in HBV‐ and HCV‐related HCC, and antiviral treatment after resection has been associated with lower recurrence and better survival [29]. In our cohort, antiviral therapy was not independently associated with recurrence, but its timing, duration, and adherence were heterogeneous, and etiology was not a covariate in the models. This may have hidden a real effect.

Clinically, GAAD uses tests that are already measured in HCC care (sex, age, AFP, and PIVKA‐II), and it could help refine surveillance intensity after resection. Unlike MVI, which is known only after pathological examination, it is available before surgery. In the era of expanding immunotherapy and targeted agents, multiple clinical trials are evaluating adjuvant treatments following curative resection for HCC [30]. Our findings suggest that GAAD could serve as one component of preoperative recurrence risk assessment, for example when considering surveillance intensity or selecting patients for adjuvant trials, together with established factors such as BCLC stage and MVI. Its practical value is limited by the cutoff since GAAD ≥ 1 classified 83.3% of patients as high‐risk, limiting its ability to single out a small high‐risk group, and it was not associated with OS. Whether it can guide adjuvant therapy remains to be shown in prospective studies.

This study has several limitations. First, its retrospective, single‐center design may introduce selection bias and limit generalizability; therefore, prospective multi‐center studies are needed to validate these findings across broader populations and healthcare settings. In addition, our analysis was limited to patients who underwent curative liver resection, and it remains unclear whether GAAD retains prognostic value in other treatment contexts such as radiofrequency ablation, liver transplantation, or systemic therapy. Second, the cutoffs for GAAD, AFP, and PIVKA‐II were derived and tested in the same cohort, which tends to overstate associations, and external validation is needed. The discriminative ability of GAAD ≥ 1 was also modest (AUC 0.675; specificity 29.4%), and 83.3% of patients were classified as high‐risk. Third, recurrences within the first 6 months were not excluded, and some of these may reflect residual disease rather than new tumors. Fourth, the OS models had limited events per variable and did not account for post‐recurrence treatment, and antiviral therapy was analyzed as a binary variable, without data on treatment duration or adherence. Fifth, the subgroup with low AFP and PIVKA‐II was small, and the subgroup analyses were not adjusted for multiple comparisons. Finally, GAAD scores were generated with the manufacturer's tool, and we could not examine its internal formula. Prospective multicenter studies are needed to validate these findings.

## 5 Conclusion

In this cohort of patients undergoing curative hepatectomy for HCC, a GAAD score ≥ 1 was independently associated with recurrence, including among patients with BCLC stage 0/A, but not with overall survival. GAAD ≥ 1 was also associated with larger tumor size and a higher rate of microvascular invasion. These exploratory findings require validation in independent cohorts before GAAD can be considered for recurrence risk assessment after resection.

## Funding

This work was supported by Chang Gung Memorial Hospital (CMRPG8P0621).

## Conflicts of Interest

The authors declare no conflicts of interest.

## Supporting information


**Supplementary Figure S1:** Recurrence‐free survival (RFS; A) and overall survival (OS; B) among patients with hepatocellular carcinoma (HCC) with low AFP (< 7 ng/mL) and low PIVKA‐II (< 22 ng/mL), stratified by GAAD score (< 1 vs. ≥ 1).

**Supplementary Table S1:** Prognostic factors associated with HCC recurrence in AFP < 7 ng/mL subgroup
**Supplementary Table S2:** Spearman correlation coefficients among gender, age, AFP, PIVKA‐II, and GAAD.
**Supplementary Table S3:** Characteristics of the HCC patients stratified by GAAD.

## Data Availability

The data that support the findings of this study are available from the corresponding author upon reasonable request.
