# Supplementary material for: GAAD Score as a Prognostic Model for Recurrence of Hepatocellular Carcinoma After Curative Resection
Source: Kaohsiung J Med Sci. 2026 Nov 14:e70318. Online ahead of print. doi: 10.1002/kjm2.70318 (PMC13768872; doi:10.1002/kjm2.70318)
Supplement: Supplementary file 1 [file KJM2-9999-e70318-s001.docx]

**Supplementary**

**Table S1. Prognostic factors associated with HCC recurrence in AFP<7 ng/mL subgroup**

| **Variable** | **Comparison** | **Univariable** | | **Multivariable 1** | | **Multivariable 2** | |
| --- | --- | --- | --- | --- | --- | --- | --- |
|  |  | **HR (95% CI)** | ***P*-value** | **HR (95% CI)** | ***P*-value** | **HR (95% CI)** | ***P*-value** |
| Age (years) | > 60 vs. ≦ 60 | 1.41 (0.85-2.35) | 0.118 |  |  | 1.35 (0.79-2.32) | 0.269 |
| Sex | Male vs. Female | 1.42 (0.75-2.71) | 0.282 |  |  | 1.55 (0.80-3.00) | 0.197 |
| Diabetes mellitus | Yes vs. No | 1.31 (0.81-2.12) | 0.263 |  |  |  |  |
| PIVKA-II (ng/mL) | ≧ 22 vs. <22 | 1.37 (0.81-2.31) | 0.243 |  |  | 1.61 (0.92-2.81) | 0.097 |
| GAAD | ≧ 1 vs. < 1 | 1.80 (1.04-3.11) | 0.034 | 2.50 (1.39-4.51) | 0.002 |  |  |
| Liver cirrhosis | Yes vs. No | 1.96 (1.21-3.17) | 0.006 | 3.28 (1.89-5.67) | <0.001 | 2.71 (1.59-4.61) | <0.001 |
| BCLC stage | B/C vs. 0/A | 2.34 (1.35-4.05) | 0.002 | 2.82 (1.51-5.25) | 0.001 | 2.70 (1.45-5.04) | 0.002 |
| Tumor size (cm) | ≧ 5 vs. ＜5 | 1.10 (0.66-1.85) | 0.715 |  |  |  |  |
| Tumor number | Multiple vs. Single | 1.06 (0.56-2.03) | 0.861 |  |  |  |  |
| MVI | Yes vs. No | 2.02 (1.26-3.23) | 0.004 | 1.61 (0.98-2.62) | 0.058 | 1.67 (1.01-2.74) | 0.044 |
| Satellite nodule | Yes vs. No | 2.25 (0.82-6.21) | 0.117 | 2.27 (0.42-3.88) | 0.667 | 0.91 (0.29-2.87) | 0.886 |
| Antiviral therapy | Yes vs. No | 0.76 (0.45-1.26) | 0.282 |  |  |  |  |

HR, hazard ratio; CI, confidence interval; AFP, alpha-fetoprotein; PIVKA-II, protein induced by vitamin K absence or antagonist II; BCLC, Barcelona clinical liver cancer; MVI, microvascular invasion

Multivariable 1: include GAAD, liver cirrhosis, BCLC stage, tumor size, tumor number, MVI, satellite nodule, and antiviral therapy.

Multivariable 2: include age, sex, diabetes mellitus, AFP, liver cirrhosis, BCLC stage, tumor size, tumor number, MVI, satellite nodule, and antiviral therapy.

**Supplementary**

**Table S2. Spearman correlation coefficients among gender, age, AFP, PIVKA-II, and GAAD**

| **Variable** | **Age** | **Gender*** | **AFP** | **PIVKA-II** | **GAAD** |
| --- | --- | --- | --- | --- | --- |
| Age | 1.000 | −0.187 | −0.161 | 0.099 | 0.156 |
| Gender* | −0.187 | 1.000 | −0.101 | 0.099 | 0.097 |
| AFP | −0.161 | −0.101 | 1.000 | 0.172 | 0.723 |
| PIVKA-II | 0.099 | 0.099 | 0.172 | 1.000 | 0.672 |
| GAAD | 0.156 | 0.097 | 0.723 | 0.672 | 1.000 |

Values are Spearman correlation coefficients (n = 294). Bold indicates a statistically significant correlation with GAAD (P < 0.05).

* Gender was coded as 0 = female and 1 = male.

GAAD, gender, age, AFP, and PIVKA-II; AFP, alpha-fetoprotein; PIVKA-II, protein induced by vitamin K absence or antagonist II.

**Supplementary**

**Table S3. Characteristics of the HCC patients stratified by GAAD**

|  | **GAAD ≥ 1**  **(n = 245)** | **GAAD < 1**  **(n = 49)** | ***P* value** |
| --- | --- | --- | --- |
| Age (years), median (IQR) | 64.0 (55.0-71.0) | 58.0 (46.1-62.6) | <0.001 |
| Male, n (%) | 194 (79.2) | 35 (71.4) | 0.232 |
| Diabetes mellitus, n (%) | 76 (31.0) | 15 (30.6) | 0.955 |
| Alcoholism, n (%) | 30 (12.2) | 5 (10.2) | 0.837 |
| CHB, n (%) | 138 (56.3) | 33 (67.3) | 0.153 |
| CHC, n (%) | 87 (35.5) | 15 (30.6) | 0.511 |
| Platelets < 150 10^3^/µL, n (%) | 90 (36.7) | 21 (42.9) | 0.420 |
| Albumin (g/dL), median (IQR) | 4.1 (3.8-4.4) | 4.1 (3.9-4.5) | 0.400 |
| Total bilirubin (mg/dL), median (IQR) | 0.7 (0.5-0.9) | 0.7 (0.5-0.8) | 0.275 |
| AFP (ng/mL) | 13.4 (3.7-228) | 3.0 (2.0-4.0) | <0.001 |
| PIVKA-II (ng/mL) | 91.6 (25.9-426) | 16.5 (14.2-23.5) | <0.001 |
| Liver cirrhosis, n (%) | 92 (37.7) | 22 (44.9) | 0.346 |
| BCLC stage, n (%) |  |  | 0.553 |
| 0 / A | 196 (80.0) | 41 (83.7) |  |
| B / C | 49 (20.0) | 8 (16.3) |  |
| Tumor size (cm)a, median (IQR) | 3.7 (2.6-5.7) | 2.4 (2.0-3.5) | <0.001 |
| Tumor number, multiple, n (%) | 38 (15.5) | 7 (14.3) | 0.828 |
| MVI, n (%) | 143 (58.4) | 15 (30.6) | <0.001 |
| Satellite nodule, n (%) | 16 (6.5) | 1 (2.0) | 0.219 |
| Hepatic steatosis, n (%) | 67 (27.7) | 15 (30.6) | 0.678 |

Data are expressed as median (interquartile range) or n (%). P values were calculated with the Mann–Whitney U test for continuous variables and the Chi-square test or Fisher’s exact test for categorical variables.

Abbreviations: CHB, chronic hepatitis B; CHC, chronic hepatitis C; AFP, alpha-fetoprotein; PIVKA-II, protein induced by vitamin K absence or antagonist II; BCLC, Barcelona clinical liver cancer; MVI, microvascular invasion

^a^ Diameter of the largest tumor nodule
